# Uterine artery embolisation: fertility, adenomyosis and size – what is the evidence?

**DOI:** 10.1186/s42155-023-00353-2

**Published:** 2023-02-27

**Authors:** Leto Mailli, Shyamal Patel, Raj Das, Joo Young Chun, Seyed Renani, Sourav Das, Lakshmi Ratnam

**Affiliations:** 1 Department of Interventional Radiology, St George’s University Hospital, London, UK; 2 Department of Obstetrics and Gynaecology, St George’s University Hospital, London, UK

**Keywords:** Uterine artery embolisation, Fertility, Pregnancy rate, Miscarriage rate, Adenomyosis, Outcomes, Re-intervention, Complications, Large fibroid volume, Giant fibroids

## Abstract

**Background:**

Uterine artery embolisation is well established as a treatment for symptomatic fibroids, however, there remain some uncertainties. We have carried out a focused literature review on three particularly challenging aspects – post-procedure fertility, symptomatic adenomyosis and large volume fibroids and uteri, to enable operators to utilise evidence-based guidance in patient selection, consent, and management.

**Review:**

Literature searches were performed of the PubMed/Medline, Google scholar, EMBASE and Cochrane databases.

The outcomes of our analysis of studies which recorded fertility rates in women desiring pregnancy following UAE for symptomatic fibroids found an overall mean pregnancy rate of 39.4%, live birth rate of 69.2% and miscarriage rate of 22%. The major confounding factor was patient age with many studies including women over 40 years who already have lower fertility compared to younger cohorts. Miscarriage rates and pregnancy rates in the studies analysed were comparable to the age matched population.

Treatment of pure adenomyosis and adenomyosis with co-existing uterine fibroids with UAE has been shown to produce symptomatic improvement with better outcomes in those with combined disease. Although the effectiveness is not as high as it is in pure fibroid disease, UAE provides a viable and safe alternative for patients seeking symptom relief and uterine preservation.

Our analysis of studies assessing the outcomes of UAE in patients with large volume uteri and giant fibroids (> 10 cm) demonstrate no significant difference in major complication rates demonstrating that fibroid size should not be a contraindication to UAE.

**Conclusion:**

Our findings suggest uterine artery embolisation can be offered to women desiring pregnancy with fertility and miscarriage rates comparable to that of the age-matched general population. It is also an effective therapeutic option for symptomatic adenomyosis as well as for the treatment of large fibroids > 10 cm in diameter. Caution is advised in those with uterine volumes greater than 1000cm^3^. It is however clear that the quality of evidence needs to be improved on with an emphasis on well-designed randomised controlled trials addressing all three areas and the consistent use of validated quality of life questionnaires for outcome assessment to enable effective comparison of outcomes in different studies.

**Supplementary Information:**

The online version contains supplementary material available at 10.1186/s42155-023-00353-2.

## Background

Uterine artery embolisation (UAE) is a safe and effective therapeutic option for symptomatic uterine fibroids (Gupta et al. 2014). Originally described for the management of post-partum haemorrhage, Ravina et al. (1995) were the first to describe UAE for the treatment of symptomatic fibroids (Ravina et al. 1995; Pelage et al. 1998). Subsequently, UAE has grown in popularity as a well-tolerated, minimally invasive alternative to existing surgical methods (Gupta et al. 2014; Pelage et al. 2000; Walker and Pelage 2002). However, there are specific aspects surrounding UAE which remain a source of debate and uncertainty.

The effect of UAE on future fertility remains controversial. Joint guidelines by the Royal College of Obstetricians and Gynaecologists and the Royal College of Radiologists of the United Kingdom (UK) (2013) acknowledge there is little high-quality evidence available to help draw conclusions in this matter. They recommend UAE should only be offered to women of childbearing age who wish to preserve fertility after an informed discussion (The Royal College of Obstetricians and Gynaecologists and the Royal College of Radiologists 2013), whereas many other international guidelines recommend avoiding UAE altogether in this cohort (Marret et al. 2012; Carranza-Mamane et al. 2015; Stokes et al. 2010). This illustrates the difficulties faced by operators in dealing with patients seeking UAE while wishing to maintain fertility.

Many patients with symptomatic adenomyosis also have uterine fibroids, and whilst there are publications outlining the use of UAE to treat these patients with encouraging results (de Bruijn et al. 2017a), the evidence for this is not as clear, or as established as it is for UAE for the treatment of fibroids alone. Finally, many operators consider the use of UAE to be relatively contraindicated in patients with large volume uteri and large fibroids > 10 cm in diameter due to perceived increased complication or failure rates (Parthipun et al. 2010).

The aim of this review is to present the evidence base for tackling these three challenging aspects surrounding UAE, to assist operators in patient selection and management and having informed discussions with clinicians and patients.

## Methods

We performed separate electronic literature searches for each topic using the Pubmed/Medline, Google scholar, Cochrane and EMBASE databases in July 2022. Medical subject headings (MeSH) and keywords (for example – ‘uterine artery embolisation’ and ‘fertility’) were utilised with searches performed in turn using each database. The reference list of relevant papers was also reviewed for additional studies. Our Pubmed search strategy is included in Additional file 1. Titles and abstracts were screened, and the remaining full text articles were reviewed by two authors for each topic, with any discrepancies resolved by discussion with a third author.

We included clinical studies with ranging levels of evidence– randomised controlled trials (RCT), controlled clinical trials (CCT), comparative studies, prospective/retrospective cohort studies, case-control studies and case series. Populations, interventions and outcomes are outlined in Table 1. The exclusion criteria included papers which were not published in English, measured different outcomes (e.g., AMH levels rather than actual pregnancy rates), single case reports, patients who underwent UAE for indications other than symptomatic fibroid disease/adenomyosis and papers in which complete outcomes/results were not available (e.g., the number of women who desired to preserve fertility was not clearly reported or if birth and miscarriage rates were not provided). Data extraction was performed on the remaining studies to identify study design, sample size, mean follow up and the desired outcomes for each research question.

**Table 1:** Population, interventions, outcomes assessed for each topic

	Fertility	Adenomyosis	Large fibroids (> 10 cm diameter +/− uterine volume > 750 cm^**3**^)
**Population**	Women desiring fertility following UAE for symptomatic fibroids	Women who underwent UAE for symptomatic adenomyosis +/− fibroids	Women with large symptomatic fibroids who underwent UAE
**Intervention**	Uterine artery embolisation with any embolic material	Uterine artery embolisation with any embolic material	Uterine artery embolisation with any embolic material
**Primary outcome**	Pregnancy rate in women who underwent UAE and expressed a desire to preserve fertility	Symptomatic improvement following UAE	Symptomatic improvement following UAE
**Secondary outcome(s)**	Percentage of live births and miscarriages	Symptomatic improvement between adenomyosis alone or adenomyosis + fibroids	Complication/re-intervention rate following UAE

Formal ethical/institutional board review is not required for this type of study.

## UAE and fertility

Following our literature search and applying our exclusion criteria, a total of 17 studies were included for further analysis, which are summarised in Table 2 (Mara et al. 2008; Daniels et al. 2021; Holub et al. 2008; Mara et al. 2012; McLucas et al. 2001; Pron et al. 2005; Kim et al. 2008; Pinto Pabón et al. 2008; McLucas 2013; Pisco et al. 2017; Torre et al. 2017; Walker and McDowell 2006; Hirst et al. 2008; Bonduki et al. 2011; Serres-Cousine et al. 2021; Ravina et al. 2000; Firouznia et al. 2009; Redecha Jr et al. 2013). Our analysis found a mean pregnancy rate of 39.4%, live birth rate of 69.2% and miscarriage rate of 22% (Table 2). One of the key factors identified during our analysis is that many studies report the number of women with a desire to preserve their fertility. This was usually assessed at the time of enrolment; however, this does not necessarily consider the number of women who were actively trying to conceive throughout the follow up period which has implications on the overall pregnancy rate.

**Table 2:** Studies of pregnancy following UAE for fibroids (Mara et al. 2008; Daniels et al. 2021; Holub et al. 2008; Mara et al. 2012; McLucas et al. 2001; Pron et al. 2005; Kim et al. 2008; Pinto Pabón et al. 2008; McLucas 2013; Pisco et al. 2017; Torre et al. 2017; Walker and McDowell 2006; Hirst et al. 2008; Bonduki et al. 2011; Serres-Cousine et al. 2021; Ravina et al. 2000; Firouznia et al. 2009)

Study, year	Study design	Comparison	Cohort size (n)	Number trying to conceive (n)	Follow up (months)	Mean age (y)	Total pregnancies (n)	Pregnancy rate (actual number of women who became pregnant/those trying to conceive)	Live births (rate)	Miscarriages (rate)
Mara et al. 2008	RCT	Myomectomy	58	26	24.9	32.2	17	13/26 (50%)	5/17 (29.4%)	9/17 (52.9%)
Daniels et al. 2021	RCT	Myomectomy	98	47	48	41	15	12/47 (25.5%)	9/15 (60%)	5/15 (33%)
Holub et al. 2008	Non-randomised controlled trial	Laparoscopic uterine artery occlusion	112	39	NS	32.3	28	20/39 (51.2%)	10/28 (35.7%)	14/25 (56%)
Mara et al. 2012	Non-randomised controlled trial	Laparoscopic uterine artery occlusion	100	42	NS	33.1	42	29/42 (69%)	23/42 (54.7%)	13/42 (31%)
McLucas et al. 2001	Prospective cohort	N/A	400	52 (< 40 years old)	16	N/S	17	14/52 (26.9%)	10/17 (58.8%)	5/17 (29.4%)
Pron et al. 2005	Prospective cohort	N/A	555	35	24	34	24	21/34 (61.8%)	18/24 (75%)	4/24 (16.7%)
Kim et al. 2008	Prospective cohort	N/A	87	19	45	36.6	15	12/19 (63.2%)	6/15 (40%)	3/15 (20%)
Pinto Pabón et al. 2008	Prospective cohort	N/A	100	57(39 patients < 40 years old)	NS	35	11	10/57 (17.5%)	8/11 (72.7%)	3/11 (27.3%)
McLucas 2013	Prospective cohort	N/A	44	40 (< 40 years old)	NS	33.4	28	22/40 (55%)	20/28 (71.4%)	3/28 (10.7%)
Pisco et al. 2017	Prospective cohort	N/A	359	359	69	35.9	182	149/359 (41.5%)	150/182 (82.4%)	23/182 (12.6%)
Torre et al. 2017	Prospective cohort	N/A	15	15	43.1	34.8	12	8/15 (53.3%)	10/12 (83.3%)	1/12 (8.3%)
Walker and McDowell 2006	Retrospective cohort	N/A	1200	108	NS	NS	56	33/108 (30.5%)	33/56 (58.9%)	17/56 (30.4%)
Hirst et al. 2008	Retrospective cohort	Hysterectomy	649	187	55.2	43.8	37	27/187 (14.4%)	19/37 (51.4%)	15/37 (40.5%)
Bonduki et al. 2011	Retrospective cohort	N/A	187	75	42	34.3	15	15/75 (20%)	14/15 (93%)	1/15 (6.7%)
Serres-Cousine et al. 2021	Retrospective cohort	N/A	398	158	NS	43	148	102/158 (64.6%)	109/148 (73.6%)	26/148 (17.6%)
Ravina et al. 2000	Case series	N/A	9	9	NS	36	12	9/9 (100%)	7/12 (58.3%)	5/12 (41.7%)
Firouznia et al. 2009	Case series	N/A	102	23	24	35.7	15	14/23 (60.9%)	13/15 (86.7%)	2/15 (13.3%)
Redecha Jr et al. 2013	Case series	N/A	98	21	48	38.7	7	6/21 (28.6%)	7/7 (100%)	0
TOTAL								516/1311 (39.4%)	471/681 (69.2%)	149/678 (22%)

*N/A* not applicable, *NS* not stated

Of the studies included in the analysis there were two RCTs which compared outcomes following UAE and myomectomy (Mara et al. 2008; Daniels et al. 2021). Mara et al. (2008) found pregnancy rates of 50% in the UAE cohort (26 patients) compared to 78% in the myomectomy (either open or laparoscopic) cohort (40 patients). However, of note, 4 patients in the UAE cohort and 1 patient in the myomectomy group had two pregnancies, which makes the cumulative pregnancy rate 65.4% in the UAE group and 80% in the myomectomy group. The authors quote an abortion rate of 64% in the UAE group. This includes 2 patients having terminations as well as 9 abortions out of 17 pregnancies. If the terminations are excluded, the abortion rate is lower at 52%. Similarly, the abortion rate for myomectomy is quoted as 23%, however the data shows 6 abortions and 2 terminations, once again if terminations are excluded, the rate of miscarriage would be 19% (6 out of 32 pregnancies). The headline figures from this paper, along with a handful of cohort studies which suggest similar obstetric complications, have largely formed the basis of guidance that UAE should not be the first line option offered to women of childbearing age (The Royal College of Obstetricians and Gynaecologists and the Royal College of Radiologists 2013; Marret et al. 2012; Carranza-Mamane et al. 2015; Stokes et al. 2010). However, the limited number of patients studied (only 26 undergoing UAE compared to 40 in the myomectomy cohort) make it difficult to extrapolate these findings to the general population or to make accurate recommendations from this data.

A more recent RCT published by Daniels et al. (2021), compares UAE and myomectomy with a 4-year follow up period. They report a cumulative pregnancy rate of 15% (compared to 6% in the myomectomy cohort). However, 42% of the total cohort were lost to follow up. Only 48% of women following UAE desired fertility (which equates to 47 patients). We calculated a pregnancy rate of 25.5%, which is lower than the rate reported by Mara et al. (2008). However, the discrepancy in mean age between the two cohorts (40.2 years – Daniels et al. (2021) compared to 32.8 years - Mara et al. (2008)) is very likely to have contributed to this. The miscarriage rate was 33%, which is much lower than that of Mara et al. (2008) and closer to the miscarriage rate of the age-matched general population.

Two prospective controlled studies compare UAE with laparoscopic uterine artery ligation (LUAO) and found pregnancy rates of 51%–69% (Holub et al. 2008; Mara et al. 2012). Miscarriage rates again were variable between 31 and 56%. However, Holub et al. (2008) noted that their UAE cohort were on average older and had larger fibroids than the LUAO cohort (32.8 years vs 30.6 years) (Holub et al. 2008; Walker and McDowell 2006). Similarly, Mara et al. (2012) UAE cohort overall had higher BMI and larger fibroids (Mara et al. 2012). Alongside these confounding factors which could themselves affect pregnancy and miscarriage rates, limited absolute numbers of pregnancies again makes drawing significant conclusions from these data difficult.

The remaining 13 studies involved 7 prospective cohorts, 4 retrospective cohorts and 3 case series. We found significant variation between these studies in pregnancy, live birth and miscarriage rates following UAE. Given the nature of the studies, they are open to significant bias. Apart from the RCT, the UAE cohorts demonstrate selection bias and may have had lower fertility potential to begin with. For example, 57 patients (14%) in the UAE cohort described by McLucas et al. (2001) had previously reported pregnancy loss and 11 patients (2.8%) underwent previous myomectomy with confirmed adhesions prior to UAE. Variation in technique was also reported by Pisco et al. (2017) who present a cohort of 359 patients undergoing UAE, all of whom had been attempting to conceive for at least 1 year, and 19.5% of whom were over 40 years of age. In 44.6% (160) of the patient cohort, partial embolisation was carried out intentionally to preserve fertility based on the assumption that conventional UAE may decrease fertility. The overall pregnancy rate for the whole cohort with up to 10 years follow up period was 42%. For 85.5% of these women, this was their first pregnancy and occurred after undergoing UAE. Patients undergoing partial UAE had similar clinical results, a lower complication rate, a higher pregnancy rate with live births and fewer obstetric complications than those undergoing conventional UAE. The authors raised the possibility of a role for UAE as a fertility restoring procedure in women with uterine fibroids (Pisco et al. 2017).

We found the most important factor affecting fertility was the variable age of patients with many studies including patients who were over the age of 40. It is well documented that advancing age as an independent factor negatively impacts fertility and increases obstetric complications such as miscarriage. Hirst et al. (2008) describe the lowest pregnancy rate of all the included studies (14.4%) however the mean age of their UAE cohort was 43.8 years (Hirst et al. 2008). Similarly, Pinto Pabón et al. 2008 report a pregnancy rate of 17.5% following UAE (Pinto Pabón et al. 2008). However only 39 of the 57 patients wishing to preserve fertility were less than 40 years of age. When calculating the pregnancy rate in their cohort in those under the age of 40 years, this improves to 23%.

The American College of Obstetricians and Gynaecologists report fecundity (the likelihood of achieving pregnancy in one menstrual cycle) in healthy women aged above 40 years as 5%. This improves to between 12.4%–22.1% in women aged 35–40 years and 31.9% in women aged under 35 years (American College of Obstetricians and Gynecologists Committee on Gynecologic Practice and Practice Committee 2014). Whilst the number of cycles was not measured, follow up ranged between 16 and 69 months and the average age was 35.9 years. Therefore, the mean pregnancy rates calculated in our analysis of approximately 39.4% following UAE would be comparable to the age-matched general population. The miscarriage rate in women aged 30–34 years is approximately 15% which increases to 24.6% in those aged 35–39, and 51% in women aged 40–44 years (Nybo Andersen et al. 2000). Thus, the calculated miscarriage rate of 22% following UAE is also comparable to that of the age-matched general population.

Ghanaati et al. (2020) performed a meta-analysis which came to similar conclusions regarding the variability and low levels of evidence surrounding fertility outcomes following UAE. As with our own analysis, they suggest the overall pooled rate of pregnancy and obstetric complications following UAE for symptomatic fibroids are comparable to the general population (Ghanaati et al. 2020) and that UAE can be offered to women of childbearing age. Nevertheless, there remains a clear need for high quality RCTs with enough patients below the age of 40 actively trying to conceive to confirm these outcomes.

## UAE and adenomyosis

Adenomyosis is often present concurrently with uterine fibroids. Given the efficacy of UAE in fibroid disease, it has also been proposed as an alternative minimally invasive treatment option for the management of adenomyosis (Taran et al. 2013; Ferenczy 1998; Antero et al. 2020; Froeling et al. 2012).

A review of UAE in adenomyosis published in 2011 by Popovic et al. (2011) reviewed studies from 1999 to 2010 and evaluated 511 women with an overall finding of symptomatic relief in 387 women (75.7%) with a median follow-up of 26.9 months. Symptom relief in the short term was seen in 83.3% with pure adenomyosis and 92.9% with combined adenomyosis and fibroids, and in the long term, symptom improvement was seen in 64.9% (pure adenomyosis) and 82.4% (combined cases). Data showed that the improvement was most dramatic in the short term (3–12 months) with trends towards long term recurrence. Although the review concluded that UAE was deemed favourable in terms of cost-effectiveness, minimal side-effects, and retention of fertility the quality of evidence was deemed insufficient to establish UAE as a potential first-line treatment for adenomyosis.

A research group in the Netherlands have continued to explore this area with a series of studies over the past decade. Smeets et al. (2012)) studied an initial cohort of 40 patients with adenomyosis, of which 18 patients had pure adenomyosis and 22 patients had both adenomyosis and fibroids. Symptoms were assessed using the UFS-QoL and found 29 patients had a good relief of symptoms and remained asymptomatic throughout the follow-up period. During the follow-up period, 7 patients (18%) underwent hysterectomy and some of these patients also showed evidence of progressive junctional zone thickness on MRI. In conclusion, the authors found that of the patients who retained their uterus, 87.9% (29 out of 33) were asymptomatic. The need for hysterectomy or clinical outcomes were not influenced by the additional presence of fibroids to adenomyosis. The presence of a thick junctional zone at baseline MRI and at 3 months follow-up after UAE is a predictive factor for the need for additional therapy (hysterectomy or repeat UAE) during long-term follow-up, which is a useful additional factor to consider when counselling patients.

These findings were similar to a prior study by Kim et al. (2007) which also found that long-term clinical success in women followed up for > 3.5 years was seen in 50 of 54 women (93%) with only 5 women (9%) in the cohort undergoing hysterectomy.

Nijenhuis et al. (2015) evaluated 29 women consecutively undergoing UAE with Polyzene-F hydrogel microspheres (Nijenhuis et al. 2015). Of these patients, 14 had pure adenomyosis and 15 had adenomyosis in combination with fibroids. At a final mean follow-up of 3 years (mean 37 months), 22 of 29 women (76%) were asymptomatic. No significant difference in outcome was seen between women with pure adenomyosis and those with additional fibroids with overall good clinical outcomes. The baseline mean symptom score was 67 out of 100. This was seen to have reduced to a mean of 22 at 3 months, and then 15 at final follow-up of 3 years. One patient had a hysterectomy at 17 months for ongoing symptoms.

A longer-term, 7-year follow-up study of this same patient cohort was then published by de Bruijn et al. (2017b). Quality of life and symptom-severity scores were collected, and evaluation performed as to whether the patient underwent menopause and or had a hysterectomy during that period. 82% (23/28) of patients avoided the need for hysterectomy in the 7-year follow up. Overall, 21/29 patients (72%) were satisfied with UAE for adenomyosis. 42% of women reported the onset of menopause. Of the 5 patients undergoing hysterectomy, 4 occurred after the initial 3 year follow up period reported in the first paper (Nijenhuis et al. 2015), underlining the importance of long-term follow-up. The median age of the patients studied in this cohort was 50 (range 36–59). The two studies demonstrated that the greatest improvement in symptom score occurred early after the UAE procedure was carried out. This improvement was then noted to be sustained after the initial period. These facts are also of value in counselling older patients who may be approaching menopause as they support the avoidance of major surgery.

Following this, a systematic review by de Bruijn et al. was published in JVIR in 2017 de Bruijn et al. 2017a) which included studies from 1999 to 2015. The evidence was divided into 4 groups as follows: 1) Pure adenomyosis; short term outcomes (< 12 months) – 9 studies reported (307 patients), 2) Adenomyosis with fibroids (combined adenomyosis); short term outcomes – 6 studies reported (141 patients), 3) Pure adenomyosis; long term outcomes (> 12 months) – 16 studies reported (430 patients) and 4) Combined adenomyosis; long term outcomes – 10 studies reported (146 patients). The studies were a mixture of mainly prospective cohort studies and fewer retrospective cohort studies. These groups showed a short-term improvement in 89.6% with pure adenomyosis and 94.3% in the combined fibroid and adenomyosis group. There was a long-term improvement in 74% with pure adenomyosis and 84.5% in the combined fibroid and adenomyosis group. Overall, there was an improvement in symptoms in 83.1% of patients.

The authors noted several difficulties related to the data as most studies incurred small sample sizes, relatively few studies used validated questionnaires such as UFS-QOL and there was variation in particles sizes used for embolisation. There were no randomised controlled trials (RCTs) available for inclusion. Of the 30 studies (22 prospective cohorts and 8 retrospective cohorts) included in the systematic review, only 6 studies assessed symptom improvement with a validated quality of life questionnaire. We have summarised these, included an additional study and excluded a paper only available in German in Table 3. Most authors agree that the systematic use of the UFS-QOL questionnaire would aid future evaluation of pooled data and improve the quality of assessment in future trials.

**Table 3:** Studies of UAE for adenomyosis where the primary outcome was assessed using a validated QOL questionnaire (Froeling et al. 2012; Smeets et al. 2012; Nijenhuis et al. 2015; de Bruijn et al. 2017a; de Bruijn et al., 2017b; Siskin et al. 2001; Millo et al. 2010)

Study, year	Study design	Period	Cohort size (n)	Embolic	Follow up (months)	Indication	Primary Outcome	Secondary Outcome	Quality score
Siskin et al. 2001	Retrospective cohort	NR	13	255–500 μm PVA	10.2	AUB, dysmenorrhea, bulk	Symptom improvement, HRQOL	JZ thickness	13
Millo et al. 2010	Prospective cohort	NR	7	300–500 μm PVA	6	AUB, dysmenorrhea, bulk	Symptom improvement, UFS-QOL	NA	15
Froeling et al. 2012	Prospective cohort	2001–2009	40	355–900 μm TGM	40	AUB, dysmenorrhea, bulk	Symptom improvement, UFS-QOL	NA	15
Smeets et al. 2012	Prospective cohort	1999–2006	40	500–700 μm TGM	65	AUB, dysmenorrhea, bulk	Symptom improvement, UFS-QOL	Uterine volume, JZ thickness, infarction	18
^a^Nijenhuis et al. 2015	Prospective cohort	2006–2010	29	500–900 μm hydrogel microspheres	37	AUB, dysmenorrhea, bulk	Symptom improvement, UFS-QOL	Uterine volume	19
^a^de Bruijn et al., 2017a, b	Prospective cohort	2006–2010	29	500–900 μm hydrogel microspheres	84	AUB, dysmenorrhea, bulk	Symptom improvement, UFS-QOL	Menopause	NR

*NR* not reported, *AUB* abnormal uterine bleeding, *HRQOL* health related quality of life, *UFS-QOL* Uterine fibroid symptom and quality of life

^a^same patient cohort with outcomes reported at 3 years and 7 years. The De Bruijn article was not included in the systematic review

Despite the growing evidence for favorable outcomes in UAE for adenomyosis, the lack of randomized controlled trials and Level 1 evidence makes it difficult to extrapolate existing data. The outcomes of the Quality of Life after Embolisation vs Hysterectomy in Adenomyosis (QUESTA) which commenced recruitment in 2015 are currently awaited (de Bruijn et al. 2018). The primary outcome of this trial is based on the HRQOL assessment with a 24 month follow up period and imaging follow up.

### UAE technique in adenomyosis

The optimal technique to perform UAE in adenomyosis is still under debate. Studies with MRI imaging follow up have shown (Popovic et al. 2011; Lohle et al. 2007) a 27%–51% reduction in uterine volume and approximately 24% reduction of junctional zone thickness is observed after successful UAE in adenomyosis. Bae et al. (Bae et al. 2015) evaluated percentage necrosis of the adenomyosis segment on MRI in 50 patients and demonstrated that those patients with less than 34.3% necrosis had a seven-fold risk of symptom recurrence, with MRI performed at baseline and 3 months post UAE and clinical follow-up to 18 months. Most studies with imaging follow up have noted that areas of devascularisation, necrosis or infarction correlate well with symptomatic improvement (de Bruijn et al., 2017a).

The size and type of particles used in UAE for adenomyosis has been variable in the literature with most studies using a range of sizes from 250 to 900 μm non-spherical polyvinyl alcohol (PVA) particles. Kim et al. 2007 suggested that the use of a combination of small (250–355 μm) followed by larger (500 to 710 μm) PVA particles for a UAE induces a significantly higher rate of necrosis in adenomyosis than using 250–355 um or 355–500 um particles alone. A subsequent publication by Kim et al. 2011, proposed the “1–2-3 protocol” which used 150–250 μm, increasing to 250–355 μm and then 355–500 μm non-spherical PVA and achieved 82.5% complete necrosis of all adenomyotic tissue in a study of 40 patients.

It has been theorized that since adenomyosis has a more diffuse and deep distribution throughout the myometrium compared to uterine fibroids, targeting the smaller myometrial arteries results in deeper penetration into the vascular plexus enabling more effective targeting of areas of adenomyosis. In studies to date, the use of medium sized particles such as tris-acryl gelatin microspheres (TAGM) sized at 500–700 um (Lohle et al. 2007), and non-spherical PVA of 355–500 um or 500–700 um reported rates of infarction of 44.1% in the TAGM group and approximately 40% in other studies. This suggests that the use of the smaller diameter particles, such as in the 1–2-3 protocol or similar, may induce greater infarction and thereby greater symptom relief, but would benefit from further evidence. The greater degree of infarction may also result in an increase in post embolisation pain. The 1–2-3 protocol technique was however not recommended by the authors in patients who desire future pregnancies as the post procedural MRI findings demonstrated marked uterine volume reduction with endometrial thinning, and endometrial atrophy is a known cause of subfertility (Tropeano et al. 2003).

## UAE and large fibroids

Some earlier reports of UAE cited as suggesting that larger fibroid size and overall uterine volume are associated with poorer clinical outcomes and increased risk of complications only describe a single case each of complication, both of single large fibroids causing septic necrosis post UAE and requiring hysterectomy (Pelage et al. 2000; Worthington-Kirsch et al. 1998).

The EMMY trial, a multicentre randomised controlled trial (RCT) of 81 patients, showed that larger uterine volume (> 500cm^3^) and larger dominant fibroid volume (> 100 cm^3^) were associated with an increased risk of major complications (Volkers et al. 2006). Closer study of the paper shows these comprised of minor increased hospital stay, readmission for pain management or antibiotic treatment for a variety of infective causes and 12 cases of fibroid expulsion were recorded in total, but no emergency hysterectomies were required.

The FIBROID registry in 2005 reported greater symptomatic improvement in smaller leiomyomas at 1 year (Spies et al. 2005). At 3 years, larger leiomyoma size remained a predictor of poorer symptomatic improvement (Goodwin et al. 2008). Although many subsequent studies have refuted these conclusions of higher complications and poorer outcomes, large fibroid size remains a somewhat contentious issue in UAE practice. Table 4 summarises the papers studied in this section.

**Table 4:** Assessment of complications post UAE in patients with large fibroids compared with a control group (Parthipun et al., 2010; Katsumori et al., 2003; Prollius et al., 2004; Firouznia et al., 2008; Choi et al., 2013; Bérczi et al., 2015; Mollier et al., 2020)

Study, year	Study design	Period	Cohort size (n)	Giant fibroids / large volume uterus (n)	Non giant fibroids (n)	Giant Fibroid size	Uterine volume	Follow up (months)	Increased risk of major complications in giant fibroid group	Increased risk of major complications in large volume uterus group
Katsumori et al., 2003	Retrospective cohort	NR	152	47	105	> 10 cm	NR	17.5	No	No
Prollius et al., 2004	Prospective cohort	NR	64	12 (18.8%)	52	NR	>780cm^3^	12	No	No
Firouznia et al., 2008	Retrospective cohort	2001–2006	101	NR	NR	NR	NR	12	No	No
Parthipun et al., 2010	Prospective cohort	2004–2008	121	30	91	> 10 cm	>750cm^3^	12	No	No
Choi et al., 2013	Retrospective cohort	2005–2011	323	63	260	> 10 cm	>700cm^3^	12–84	No	No
Berczi et al., 2015	Retrospective cohort	2008–2012	303	41	262	> 10 cm	NR	8	No	No
Mollier et al., 2020	Retrospective cohort	2013–2018	333	NR	NR	> 10 cm	> 1000 cm^3^	NR	No	Yes

*UAE* uterine artery embolisation, *NR* not reported

In contrast to these early reports, a retrospective analysis of 152 patients showed that women with larger fibroids (> 10 cm) experienced less symptomatic improvement at 1 year but this difference did not persist at 2 years (Katsumori et al. 2003). The same study also demonstrated no increased risk of complications based on fibroid size. Similarly, a study of 61 patients showed that larger uterine volume (> 780 cm^3^) is not associated with reduced symptomatic improvement or higher rate of complications at 12 months (Prollius et al. 2004). Furthermore, a retrospective analysis of 101 patients found no correlation between size of the dominant fibroid and clinical effectiveness or complication rate at 1 year (Firouznia et al. 2008).

Subsequently, further publications echoed the findings of these studies that refuted the exclusion of patients based on fibroid and uterine volume. A prospective case control study of 121 women compared outcomes of patients grouped according to diameter of largest fibroid and uterine volume. The authors found no increased incidence in complications in women with large-diameter fibroids (> 10 cm) or large uterine volumes (>750cm^3^) at 12 months (Parthipun et al. 2010). Similarly, a retrospective analysis of 71 patients with large fibroid burden (dominant fibroid > 10 cm +/− uterine volume > 700 cm^3^) showed satisfactory imaging outcomes in terms of fibroid volume reduction and infarction rate with no incidence of severe complications at 4 years (Smeets et al. 2010). The authors concluded that large fibroid burden should not be considered a contraindication for UAE. Of note however, the authors reported 10 patients (14%) underwent subsequent hysterectomy for inadequate symptom control, suggesting clinical improvement may be somewhat limited in this group of patients.

One of the largest retrospective comparative studies involving 323 patients compared UAE outcomes based on dominant tumour dimension and uterine volume. There were no differences in outcome including volume reduction of uterus or dominant tumour, infarction rate of dominant tumour, or symptom scores at 3 months and beyond 12 months. Similarly, no differences were found in major complications and the authors concluded that outcomes in large fibroid tumours are comparable to those in smaller tumours and without an increased risk of significant complications (Choi et al. 2013). A similarly large retrospective analysis of 303 women compared two groups based on the diameter of the dominant fibroid (< or > 10 cm). Clinical effectiveness and incidence of complications were similar in both groups, although the mean follow-up was somewhat limited at less than 8 months (Bérczi et al. 2015).

Interestingly, the two most recent studies on fibroid size suggest an increased risk of complications in women with greater fibroid burden. A large retrospective case control study reviewed 333 patients with respect to intra-uterine infection post UAE and identified large uterine volume > 1000 cm^3^ to be a risk factor for infective complications although the statistically significance was relatively low (*p* = 0.049, OR 2.94 [1.15–7.54] (Mollier et al. 2020). A systematic review and meta-analysis of four retrospective cohort studies (Katsumori et al. 2003; Prollius et al. 2004; Choi et al. 2013; Bérczi et al. 2015) was carried out incorporating total of 839 total patients. There was a greater prevalence of major complications (*p* < 0.01, OR 4.7 [1.5–14.6]) and re-interventions (*p* < 0.01, OR 3.6 [1.7–7.5]) in ‘giant’ uterine fibroids, defined as dominant fibroid diameter > 10 cm +/− uterine volume > 700 cm^3^. The major complications related to fibroid expulsion, uterine infection and one patient with sexual dysfunction post UAE.

The data supports the effectiveness of the technique as a viable treatment option with appropriate counselling in this patient group. The potential for described complications is higher, and more rigorous follow up of patients and closer collaboration with gynaecology colleagues would be recommended to anticipate and deal with potential complications promptly. This includes expedient management of infections and transvaginal resection of devascularised fibroids in an elective manner to avoid the need for emergency surgery (Llewellyn et al. 2020).

## Summary

Our review found pooled rates of pregnancy of 39.4%, live birth of 69.2% and miscarriage of 22% following UAE. Given the mean age of women included in the analysis was 35.9 years, these rates are comparable to the age-matched background population and we suggest that UAE should be considered in younger patients who desire to preserve fertility.

Following UAE in the context of adenomyosis, we found an overall rate of symptomatic relief of 83% with no major differences in outcome between patients with pure adenomyosis and adenomyosis combined with fibroids. UAE is a viable alternative to hysterectomy in achieving symptomatic relief while seeking uterus preservation and fertility potential. In particular, data suggests there is good short-term improvement - an important consideration in patients approaching menopause.

We found limited early evidence reporting UAE in large fibroids (> 10 cm or uterine volume > 700 cm^3^) to be less effective and associated with increased complication rates. More recent data is contrasting, confirming that UAE is effective in treating the symptoms of uterine fibroids in large volume uteri. However, there is some evidence to suggest a higher risk of infective complications in patients with a significantly larger fibroid burden (uterine volume > 1000 cm^3^) as well as a higher rate of reintervention. Therefore, although UAE should not be contraindicated in this group, patients should be counselled appropriately prior to treatment.

## Conclusion

The available evidence supports the use of UAE as a viable management option in women seeking to preserve fertility, those with symptomatic adenomyosis and those with large fibroids.

However, there remains alack of high-quality robust data addressing these three areas. Whilst well designed randomised controlled trials remain the gold standard of evidence, the consistent use of validated quality of life questionnaires for outcome assessment in studies would enable effective comparison of outcomes from different studies. In the area of adenomyosis, the results of the QUESTA trial are awaited, which may address the issue of lack of RCT data. From a practical perspective, Standards of Practice documents from Interventional Radiology societies should also be updated to reflect the growing role, indications and suitability of UAE in these difficult clinical areas.

## Supplementary Information

**Additional file 1.** Pubmed search terms used for each outcome which were then adapted for each database.

## Data Availability

The datasets used and/or analysed during the current study are available from the corresponding author on reasonable request.

## Abbreviations

UAE: Uterine artery embolisation
LUAO: Laparoscopic uterine artery ligation
UFS-QOL: Uterine Fibroid Symptom and Quality of Life
HR-QOL: Health Related Quality of Life
SS: Symptoms Severity
TAGM: tris-acryl gelatin microspheres
PVA: polyvinyl alcohol
RCT: randomised controlled trial
