# Supplementary material for: Uterine artery embolisation: fertility, adenomyosis and size – what is the evidence?
Source: CVIR Endovasc. 2023 Feb 27;6:8. doi: 10.1186/s42155-023-00353-2 (PMC9971423; doi:10.1186/s42155-023-00353-2)
Supplement: Supplementary file 1 — Additional file 1. Pubmed search terms used for each outcome which were then adapted for each database. [file 42155_2023_353_MOESM1_ESM.docx]

**Additional file 1**

Pubmed search terms:

1. UAE and effects on fertility (pregnancy, live births and miscarriage rates vs myomectomy):

Keywords: Uterine artery embolisation, Uterine fibroid embolisation, fertility, pregnancy

Mesh: "Leiomyoma/therapy"[MAJR], "Pregnancy Complications, "Pregnancy Outcome"[MeSH]), "Uterine Artery Embolization/adverse effects"[MAJR])

Search terms: “Uterine artery emboli*”[tiab] OR “Uterine fibroid emboli*”[tiab] AND fertility[tiab]

1. UAE and adenomyosis (symptom improvement +/- reintervention rate; and adenomyosis +/- fibroids):

Keywords: Uterine artery embolisation, Uterine fibroid embolisation, adenomyosis, leiomyoma, outcomes

Mesh: "Adenomyosis/therapy"[MAJR]), "Leiomyoma/therapy"[MeSH]), "Uterine Artery Embolization/methods"[MAJR]), "Quality of Life/psychology"[MAJR]), "Treatment Outcome"[MeSH])

Search terms: “Uterine artery emboli*”[tiab] OR “Uterine fibroid emboli*”[tiab] AND adenomyo*[tiab]

1. UAE and size (giant fibroids) – (symptom improvement, complication and re-intervention rates):

Keywords: Uterine artery embolisation, Uterine fibroid embolisation, large uterus, giant fibroids, outcomes

Mesh: "Leiomyoma/therapy"[MAJR], "Uterine Artery Embolization/adverse effects"[MAJR]), "Uterine Artery Embolization/methods"[MAJR]), "Tumor Burden"[MAJR], "Quality of Life/psychology"[MAJR]) OR "Treatment Outcome"[MeSH])

Search terms: "Uterine artery emboli*"[tiab] OR "Uterine fibroid emboli*"[tiab] AND "giant fibroid"[ti] OR "large uterus"[ti]

**Additional file 1:** Pubmed search terms used for each outcome which were then adapted for each database.
